# Synthesis and crystal structure of 4-fluoro­benzyl­ammonium di­hydrogen phosphate, [FC_6_H_4_CH_2_NH_3_]H_2_PO_4_

**DOI:** 10.1107/S2056989016018090

**Published:** 2016-11-15

**Authors:** Ali Rayes, Ahlem Dadi, Najla Mahbouli Rhouma, Francesco Mezzadri, Gianluca Calestani

**Affiliations:** a Unité de Recherche, Catalyse et Matériaux pour l’Environnement et les Procédés, URCMEP, (UR11ES85), Faculté des Sciences de Gabès, Campus Universitaire, 6072 Gabès, Tunisia; b Laboratoire des Sciences des Matériaux et d’Environnement, Faculté des Sciences, Université de Sfax, BP 1171, Route de Soukra, 3018 Sfax, Tunisia; c Dipartimento di Chimica, Universitá di Parma, Parco Area delle Scienze 17A, I-43124 Parma, Italy

**Keywords:** crystal structure, organic inorganic hybrid materials, hydrogen bonds

## Abstract

The asymmetric unit of the title salt, [*p*-FC_6_H_4_CH_2_NH_3_]^+^·H_2_PO_4_^−^, contains one 4-fluoro­benzyl­ammonium cation and one di­hydrogen phosphate anion. In the crystal, the H_2_PO_4_^−^ anions are linked by O—H⋯O hydrogen bonds to build corrugated layers extending parallel to the *ab* plane. The FC_6_H_4_CH_2_NH_3_^+^ cations lie between these anionic layers to maximize the electrostatic inter­actions and are linked to the H_2_PO_4_^−^ anions through N—H⋯O hydrogen bonds, forming a three-dimensional supra­molecular network. Two hydrogen atoms belonging to the di­hydrogen phosphate anion are statistically occupied due to disorder along the OH⋯HO direction.

## Abstract 2

The crystal structure of 4-fluoro­benzyl­ammonium di­hydrogen phosphate, [FC_6_H_4_CH_2_NH_3_]H_2_PO_4_, consists of layers of 4-fluoro­benzyl­ammonium cations and di­hydrogen phosphate anions that alternate along the *c* axis, connected by hydrogen bonds into a three-dimensional network.

## Chemical context   

A hybrid compound is a material that involves both organic and inorganic components blended in the solid state on the mol­ecular scale. Such materials allow the combination of the intended properties of both the organic and inorganic components when they self-assemble in the crystal. The resulting properties do not simply consist of the sum of the individual contributions, since they also strongly depend on the nature of the inter­actions established by the different components within the structure. The nature of the inter­actions has been used to divide organic–inorganic hybrid materials into two different classes, both of them being of technological inter­est. In class I, organic and inorganic components are connected together through strong chemical covalent or iono-covalent bonds; in class II, the two components are assembled by weaker inter­actions, such as hydrogen bonds and/or van der Waals and Coulombic inter­actions.

In particular, in considering hybrid systems belonging to class II, derivatives from ortho­phospho­ric acid (H_3_PO_4_) are often associated with functionalized organic mol­ecules (amines or amides) to produce organic–inorganic materials with potentially forceful hydrogen-bonding inter­actions between donor (*D*) and acceptor (*A*) components. Among these hybrid phosphates, the di­hydrogen phosphates have received great inter­est over recent years. Indeed, these compounds can be considered the most stable organic phosphates and also the first to be studied in more detail. They have a technological inter­est in many realms, such as magnetism, electricity, optics and in biomaterials research (Adams, 1977 ▸; Hearn & Bugg, 1972 ▸).

In these compounds, the acidic di­hydrogen phosphate anion H_2_PO_4_^−^, through the formation of O—H⋯O hydrogen bonds, gives rise to various topologies of anionic substructures. In the crystal structure of 2-ammonium­benzamide di­hydrogen phosphate (Belghith *et al.*, 2015 ▸), the H_2_PO_4_^−^ tetra­hedra are associated in pairs, forming centrosymmetric finite units, while in 2,3-di­methyl­anilinium di­hydrogen phosphate (Rayes *et al.*, 2004 ▸), they form a network composed of hydrogen-bonded chains. Two-dimensional anionic layers are observed in 4-chloro­anilinium di­hydrogen phosphate (Dhaouadi *et al.*, 2008 ▸) and in 2-methyl­piperazinediium di­hydrogen phosphate (Choudhury *et al.*, 2000 ▸), while in the crystal structure of imidazolium di­hydrogen phosphate (Blessing *et al.*, 1986 ▸), the H_2_PO_4_^−^ anions are linked by hydrogen bonds to form a three-dimensional cage-type network, inside which the cations are trapped. The varieties of the observed arrangements suggest that selected packing architectures can be designed by choosing an appropriate amine.
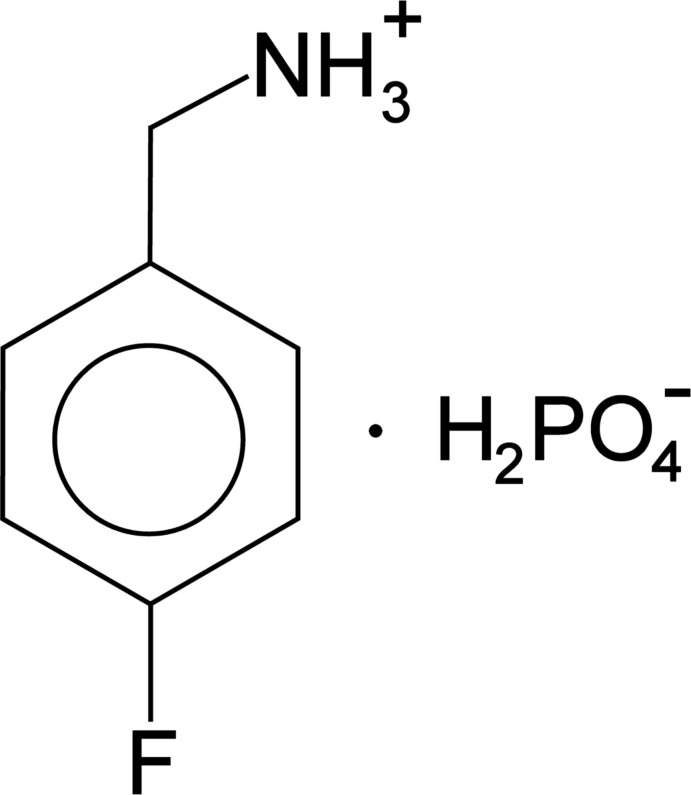


In order to enrich the knowledge of such kinds of hybrid materials and to investigate the effect of hydrogen bonds on chemical and structural features, we report here synthesis and crystal structure analysis of the novel organic di­hydrogen phosphate, (*p*-FC_6_H_4_CH_2_NH_3_)^+^·H_2_PO_4_^−^.

## Structural commentary   

**Figure 1:**
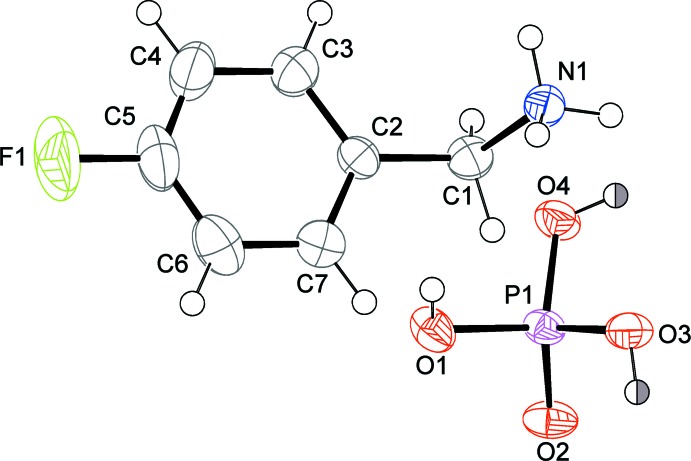
The asymmetric unit of the title compound, with displacement ellipsoids drawn at the 50% probability level. The two half-filled H atoms have a site-occupation factor of 0.5.

The title hybrid salt crystallizes in the *Pbcn* space group with one *para*-fluoro­benzyl­ammonium cation and one di­hydrogen phosphate anion in the asymmetric unit (Fig. 1 ▸). Analysis of the P—O bond lengths clearly reveals the double-bond character of the P—O2 inter­action [1.492 (4) Å], suggesting at the same time the possible protonation of the remaining O atoms showing longer bonds [P1—O1 = 1.561 (4), P1—O3 = 1.543 (4) and P1—O4 = 1.535 (4) Å]. This is confirmed by the presence of electron density peaks close to these oxygen atoms, compatible in terms of height and distance from hydrogen atoms. However, the refinement showed half occupancy for two of the three hydrogen atoms, in agreement with charge neutrality and geometric considerations (both are disordered over two positions along the O–H⋯H–O direction involving the same oxygen atom in two adjacent anions). This explains the shorter P—O3 and P—O4 bond lengths, when compared with P1—O1, revealing at the same time the composition of the resulting anion as H_2_PO_4_^−^. The organic cation exhibits a regular configuration, with distances and angles in accordance to literature data (Wang *et al.*, 2015 ▸; Klapötke *et al.*, 2003 ▸).

## Supra­molecular features   

**Figure 2:**
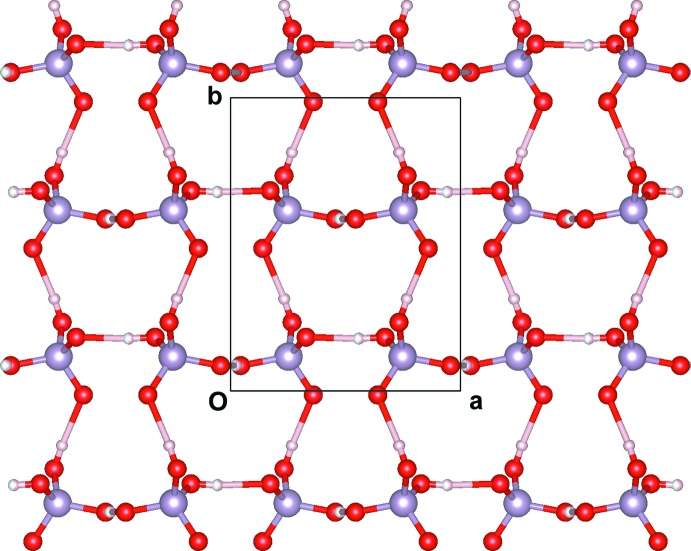
A layer of H_2_PO_4_^−^ anions, parallel to the *ab* plane, formed by hydrogen bonds displaying 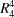
(16) graph-set ring motifs.

**Figure 3:**
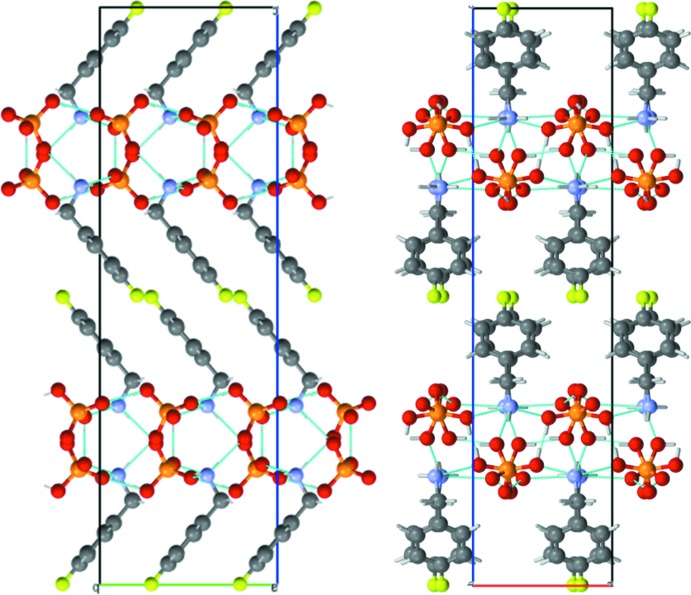
Projections of the [FC_6_H_4_CH_2_NH_3_]H_2_PO_4_ structure along the *a* axis (left) and the *b* axis (right), showing the alternate stacking of inorganic and organic layers along the *c* axis.

The presence in the title compound of a number of donor and acceptor sites leads to the formation of a complex O—H⋯O and N—H⋯O hydrogen-bonding system (Table 1 ▸) which, supported by electrostatic and van der Waals inter­actions, gives rise to the formation of a stable three-dimensional supra­molecular network. O1—H1*O*⋯O2, O3—H3*O*⋯O3 and O4—H4*O*⋯O4 hydrogen bonds connect each di­hydrogen phosphate unit to an adjacent one, which results in the formation of an infinite two-dimensional corrugated layer of anions extending parallel to the *ab* plane (Fig. 2 ▸). In the inorganic supra­molecular layers, rings with a graph-set ring motif (Etter, 1990 ▸) of 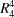
(16) are found, lying at *z* ∼1/4 and 3/4. The 4-fluoro­benzyl­ammonium cations are trapped between the anionic layers to maximize the electrostatic inter­actions and are linked to the H_2_PO_4_^−^ anions through N1—H1*A*⋯O2, N1—H1*B*⋯O3 and N1—H1*C*⋯O4 hydrogen bonds, forming 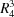
(12) graph-set motifs with the O—H⋯O bonds. The cations are anchored on both sides of the H_2_PO_4_^−^ anionic layer, resulting in the stacking of an alternating organic–inorganic supra­molecular network (Fig. 3 ▸) along the *c* axis. Within the organic network, the dipolar character of the 4-fluoro­benzyl­ammonium mol­ecule leads to an alternating anti­parallel mol­ecular stacking along the *a* axis that prevents significant π–π inter­actions between the aromatic rings but promotes van der Waals inter­actions as the unique inter­molecular inter­actions between the organic mol­ecules.

## Database survey   

A search of the Cambridge Structural Database (Version 5.37; last update February 2016; Groom *et al.*, 2016 ▸) for related compounds showed that [FC_6_H_4_CH_2_NH_3_]·H_2_PO_4_, is isotypic with 4-chloro­benzyl­ammonium di­hydrogen phosphate (Dhaouadi *et al.*, 2005 ▸). The main difference concerns the hydrogen atoms of the di­hydrogen phosphate anion. These, ordered on two sites in the latter structure, are located over three positions for the title structure, two of which show half occupancy. In spite of this difference, the resulting anionic framework and the linking of the cations are analogous in both cases. A similarly organized anionic layer is formed by self-assembly of H_2_PO_4_^−^ units in the structure of octane-1,8-di­ammonium bis­(di­hydrogen phosphate) (Mrad *et al.*, 2011 ▸). Although the amine used is of different nature, the compound crystallizes in the same space group *Pbcn* and, approximately similar to the present case, two hydrogen atoms were found to be shared along the O—H—O bonding direction involving two H_2_PO_4_^−^ groups. The difference in the organic moiety is reflected in a different anchoring of the cations on the anionic layers, building in this case a three-dimensional hydrogen-bonded network.

## Synthesis and crystallization   

Crystals of the title compound were grown by dissolving in water *p*-fluoro­benzyl­amine (purity 99%, Sigma–Aldrich) and ortho­phospho­ric acid (85%_wt_, *d* = 1.7 kg cm^−3^) in a 1:1 molar ratio. The resulting mixture was heated slightly (330 K) under constant stirring for 3 h to obtain a clear solution. Schematic­ally the reaction can be written as follows:

F(C_6_H_4_)CH_2_NH_2_ + H_3_PO_4_ → [FC_6_H_4_CH_2_NH_3_]·H_2_PO_4_

The solution thus obtained was placed in a Petri dish and kept for crystallization at room temperature without disturbance. Single crystals of the title compound, suitable for X-ray diffraction analysis, were obtained after one week (yield 82%).

## Refinement   

Crystal data, data collection and structure refinement details are summarized in Table 2 ▸. The H atoms were located in a difference Fourier map and refined as riding, with O—H = 0.82 Å, N—H = 0.89 Å, C—H = 0.93 and 0.97 Å. A rotating model was used for the OH and ammonium groups. The di­hydrogen phosphate H atoms were refined with *U*_iso_(H) = 1.5*U*_eq_(O), those of the ammonium H atoms with *U*_iso_(H) = 1.5*U*_eq_(N), and the remaining ones with *U*_iso_(H) = 1.2*U*_eq_(C). Two H atoms were found to be disordered over two positions along the O—H⋯H—O direction involving the same oxygen atom in two adjacent anions and refined with half occupancy. An outlier (524) was omitted in the last cycles of the refinement.

## Supplementary Material

Crystal structure: contains datablock(s) I. DOI: 10.1107/S2056989016018090/wm5335sup1.cif

Structure factors: contains datablock(s) I. DOI: 10.1107/S2056989016018090/wm5335Isup2.hkl

Supporting information file. DOI: 10.1107/S2056989016018090/wm5335Isup3.cml

CCDC reference: 1516161

Additional supporting information: crystallographic information; 3D view; checkCIF report

## Figures and Tables

**Table 1:** Hydrogen-bond geometry (Å, °)

*D*—H⋯*A*	*D*—H	H⋯*A*	*D*⋯*A*	*D*—H⋯*A*
O1—H1*O*⋯O2^i^	0.82	1.75	2.569 (5)	172
O3—H3*O*⋯O3^ii^	0.82	1.67	2.483 (5)	168
O4—H4*O*⋯O4^iii^	0.82	1.71	2.523 (5)	174
N1—H1*A*⋯O2^iv^	0.89	1.91	2.785 (6)	169
N1—H1*B*⋯O3^v^	0.89	1.96	2.831 (6)	167
N1—H1*C*⋯O4	0.89	2.03	2.900 (6)	164

Symmetry codes: (i) 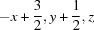; (ii) 
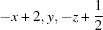
; (iii) 
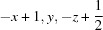
; (iv) 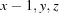; (v) 
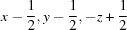
.

**Table 2:** Experimental details

Crystal data	
Chemical formula	C_7_H_9_FN^+^·H_2_PO_4_^−^
*M*_r_	223.14
Crystal system, space group	Orthorhombic, *P* *b* *c* *n*
Temperature (K)	294
*a*, *b*, *c* (Å)	7.1630 (8), 9.1309 (10), 29.694 (3)
*V* (Å^3^)	1942.1 (4)
*Z*	8
Radiation type	Mo *K*α
μ (mm^−1^)	0.29
Crystal size (mm)	0.36 × 0.31 × 0.27

Data collection	
Diffractometer	Bruker SMART CCD
Absorption correction	Multi-scan (*SADABS*; Bruker, 2008 ▸)
*T*_min_, *T*_max_	0.813, 0.846
No. of measured, independent and observed [*I* > 2σ(*I*)] reflections	19365, 1803, 1780
*R*_int_	0.031
(sin θ/λ)_max_ (Å^−1^)	0.606

Refinement	
*R*[*F* ^2^ > 2σ(*F* ^2^)], *wR*(*F* ^2^), *S*	0.077, 0.170, 1.33
No. of reflections	1803
No. of parameters	131
H-atom treatment	H-atom parameters constrained
Δρ_max_, Δρ_min_ (e Å^−3^)	0.52, −0.65

Computer programs: *APEX2* and *SAINT* (Bruker, 2008 ▸), *SHELXT* (Sheldrick, 2015*a* ▸), *SHELXL2014* (Sheldrick, 2015*b* ▸), *ORTEP-3 for Windows* (Farrugia, 2012 ▸) and *VESTA* (Momma & Izumi, 2011 ▸).

## supplementary crystallographic information

### Crystal data

**Table:**

C_7_H_9_FN^+^·H_2_PO_4_^−^	*D*_x_ = 1.526 Mg m^−^^3^
*M**_r_* = 223.14	Mo *K*α radiation, λ = 0.71073 Å
Orthorhombic, *P**b**c**n*	Cell parameters from 4573 reflections
*a* = 7.1630 (8) Å	θ = 5.5–37.8°
*b* = 9.1309 (10) Å	µ = 0.29 mm^−^^1^
*c* = 29.694 (3) Å	*T* = 294 K
*V* = 1942.1 (4) Å^3^	Prism, colourless
*Z* = 8	0.36 × 0.31 × 0.27 mm
*F*(000) = 928	

### Data collection

**Table:**

Bruker SMART CCD diffractometer	1780 reflections with *I* > 2σ(*I*)
ω scan	*R*_int_ = 0.031
Absorption correction: multi-scan (*SADABS*; Bruker, 2008)	θ_max_ = 25.5°, θ_min_ = 1.4°
*T*_min_ = 0.813, *T*_max_ = 0.846	*h* = −8→8
19365 measured reflections	*k* = −11→11
1803 independent reflections	*l* = −35→35

### Refinement

**Table:**

Refinement on *F*^2^	0 restraints
Least-squares matrix: full	Hydrogen site location: inferred from neighbouring sites
*R*[*F*^2^ > 2σ(*F*^2^)] = 0.077	H-atom parameters constrained
*wR*(*F*^2^) = 0.170	*w* = 1/[σ^2^(*F*_o_^2^) + 11.9717*P*] where *P* = (*F*_o_^2^ + 2*F*_c_^2^)/3
*S* = 1.33	(Δ/σ)_max_ < 0.001
1803 reflections	Δρ_max_ = 0.52 e Å^−^^3^
131 parameters	Δρ_min_ = −0.65 e Å^−^^3^

### Special details

**Table:**

Geometry. All esds (except the esd in the dihedral angle between two l.s. planes) are estimated using the full covariance matrix. The cell esds are taken into account individually in the estimation of esds in distances, angles and torsion angles; correlations between esds in cell parameters are only used when they are defined by crystal symmetry. An approximate (isotropic) treatment of cell esds is used for estimating esds involving l.s. planes.

### Fractional atomic coordinates and isotropic or equivalent isotropic displacement parameters (Å^2^)

**Table:**

	*x*	*y*	*z*	*U*_iso_*/*U*_eq_	Occ. (<1)
P1	0.7518 (2)	0.61709 (14)	0.29940 (4)	0.0247 (3)	
O1	0.7675 (6)	0.7342 (4)	0.33752 (11)	0.0346 (9)	
H1O	0.7332	0.8139	0.3279	0.052*	
O2	0.8598 (5)	0.4862 (4)	0.31440 (13)	0.0328 (9)	
O3	0.8281 (5)	0.6833 (4)	0.25529 (12)	0.0325 (9)	
H3O	0.9425	0.6786	0.2553	0.049*	0.5
O4	0.5439 (5)	0.5863 (4)	0.29115 (12)	0.0336 (9)	
H4O	0.5230	0.5869	0.2640	0.050*	0.5
N1	0.2305 (6)	0.3951 (5)	0.31025 (13)	0.0304 (10)	
H1A	0.1169	0.4344	0.3092	0.046*	
H1B	0.2441	0.3322	0.2876	0.046*	
H1C	0.3158	0.4655	0.3079	0.046*	
C1	0.2552 (9)	0.3166 (6)	0.35386 (16)	0.0334 (12)	
H1D	0.3705	0.2609	0.3531	0.040*	
H1E	0.1530	0.2482	0.3580	0.040*	
C2	0.2606 (8)	0.4220 (6)	0.39291 (16)	0.0297 (11)	
C3	0.0985 (9)	0.4714 (7)	0.4122 (2)	0.0427 (15)	
H3	−0.0159	0.4388	0.4013	0.051*	
C4	0.1037 (11)	0.5698 (8)	0.4480 (2)	0.0541 (19)	
H4	−0.0059	0.6043	0.4610	0.065*	
C5	0.2724 (12)	0.6139 (8)	0.46325 (19)	0.0543 (18)	
C6	0.4362 (11)	0.5685 (8)	0.4453 (2)	0.0558 (19)	
H6	0.5498	0.6013	0.4566	0.067*	
C7	0.4285 (9)	0.4709 (7)	0.4093 (2)	0.0442 (15)	
H7	0.5388	0.4383	0.3961	0.053*	
F1	0.2783 (8)	0.7094 (5)	0.49869 (14)	0.0887 (17)	

### Atomic displacement parameters (Å^2^)

**Table:**

	*U*^11^	*U*^22^	*U*^33^	*U*^12^	*U*^13^	*U*^23^
P1	0.0248 (6)	0.0201 (6)	0.0293 (6)	0.0016 (5)	−0.0057 (6)	0.0010 (5)
O1	0.043 (2)	0.0255 (18)	0.0348 (19)	0.0029 (18)	−0.0126 (19)	−0.0025 (16)
O2	0.0263 (19)	0.0222 (17)	0.050 (2)	0.0002 (16)	−0.0069 (18)	0.0051 (17)
O3	0.0229 (18)	0.042 (2)	0.0326 (19)	0.0039 (18)	0.0007 (16)	0.0095 (17)
O4	0.0226 (18)	0.048 (2)	0.0299 (19)	−0.0048 (18)	−0.0031 (16)	0.0030 (18)
N1	0.029 (2)	0.032 (2)	0.030 (2)	−0.001 (2)	0.001 (2)	−0.0028 (19)
C1	0.037 (3)	0.027 (3)	0.036 (3)	0.004 (3)	−0.002 (3)	0.002 (2)
C2	0.032 (3)	0.029 (3)	0.028 (2)	−0.001 (2)	−0.003 (2)	0.003 (2)
C3	0.039 (3)	0.050 (4)	0.040 (3)	−0.001 (3)	0.003 (3)	−0.003 (3)
C4	0.063 (5)	0.061 (4)	0.039 (4)	0.013 (4)	0.009 (3)	−0.006 (3)
C5	0.082 (5)	0.048 (4)	0.033 (3)	0.003 (4)	−0.006 (4)	−0.010 (3)
C6	0.060 (5)	0.060 (4)	0.047 (4)	−0.011 (4)	−0.013 (4)	−0.009 (3)
C7	0.042 (3)	0.050 (4)	0.041 (3)	0.007 (3)	−0.004 (3)	−0.003 (3)
F1	0.127 (4)	0.084 (3)	0.055 (2)	0.002 (3)	−0.010 (3)	−0.039 (2)

### Geometric parameters (Å, º)

**Table:**

P1—O2	1.492 (4)	C1—H1E	0.9700
P1—O4	1.535 (4)	C2—C3	1.371 (8)
P1—O3	1.543 (4)	C2—C7	1.372 (8)
P1—O1	1.561 (4)	C3—C4	1.391 (9)
O1—H1O	0.8200	C3—H3	0.9300
O3—H3O	0.8200	C4—C5	1.352 (11)
O4—H4O	0.8200	C4—H4	0.9300
N1—C1	1.491 (6)	C5—C6	1.354 (11)
N1—H1A	0.8900	C5—F1	1.367 (7)
N1—H1B	0.8900	C6—C7	1.393 (9)
N1—H1C	0.8900	C6—H6	0.9300
C1—C2	1.508 (7)	C7—H7	0.9300
C1—H1D	0.9700		
			
O2—P1—O4	113.9 (2)	H1D—C1—H1E	108.0
O2—P1—O3	112.5 (2)	C3—C2—C7	119.2 (5)
O4—P1—O3	106.3 (2)	C3—C2—C1	120.7 (5)
O2—P1—O1	107.1 (2)	C7—C2—C1	120.2 (5)
O4—P1—O1	108.1 (2)	C2—C3—C4	120.6 (6)
O3—P1—O1	108.8 (2)	C2—C3—H3	119.7
P1—O1—H1O	109.5	C4—C3—H3	119.7
P1—O3—H3O	109.5	C5—C4—C3	118.2 (7)
P1—O4—H4O	109.5	C5—C4—H4	120.9
C1—N1—H1A	109.5	C3—C4—H4	120.9
C1—N1—H1B	109.5	C4—C5—C6	123.4 (6)
H1A—N1—H1B	109.5	C4—C5—F1	118.4 (7)
C1—N1—H1C	109.5	C6—C5—F1	118.2 (7)
H1A—N1—H1C	109.5	C5—C6—C7	117.7 (7)
H1B—N1—H1C	109.5	C5—C6—H6	121.2
N1—C1—C2	111.4 (4)	C7—C6—H6	121.2
N1—C1—H1D	109.4	C2—C7—C6	121.0 (6)
C2—C1—H1D	109.4	C2—C7—H7	119.5
N1—C1—H1E	109.4	C6—C7—H7	119.5
C2—C1—H1E	109.4		

### Hydrogen-bond geometry (Å, º)

**Table:**

*D*—H···*A*	*D*—H	H···*A*	*D*···*A*	*D*—H···*A*
O1—H1*O*···O2^i^	0.82	1.75	2.569 (5)	172
O3—H3*O*···O3^ii^	0.82	1.67	2.483 (5)	168
O4—H4*O*···O4^iii^	0.82	1.71	2.523 (5)	174
N1—H1*A*···O2^iv^	0.89	1.91	2.785 (6)	169
N1—H1*B*···O3^v^	0.89	1.96	2.831 (6)	167
N1—H1*C*···O4	0.89	2.03	2.900 (6)	164

Symmetry codes: (i) −*x*+3/2, *y*+1/2, *z*; (ii) −*x*+2, *y*, −*z*+1/2; (iii) −*x*+1, *y*, −*z*+1/2; (iv) *x*−1, *y*, *z*; (v) *x*−1/2, *y*−1/2, −*z*+1/2.
